# Intravascular histiocytosis: case report of a rare disease probably associated with silicone breast implant^☆^^☆☆^

**DOI:** 10.1016/j.abd.2019.04.016

**Published:** 2020-03-19

**Authors:** Yasmin Gama Abuawad, Ticiana de Andrade Castelo Branco Diniz, Priscila Kakizaki, Neusa Yuriko Sakai Valente

**Affiliations:** Department of Dermatology, Hospital do Servidor Público Estadual de São Paulo, São Paulo, SP, Brazil

**Keywords:** Cell proliferation, Histiocytosis, Immunohistochemistry, Prostheses and implants

## Abstract

Intravascular histiocytosis is a rare condition characterized by the aggregate of histiocytes within dilated dermal vessels. The diagnosis is mainly histophatological and immunohistochemical. We describe a case of a 55 year-old female patient presenting erythematous/purple patches on the breasts, back and limbs. She previously presented ductal carcinoma in the right breast in 2006 which was treated with mastectomy and proceeded to silicone breast implant in 2009. Clinical hypothesis was telangiectatic metastatic carcinoma. Histopathology showed vascular ectasia, thrombosis and recanalization of upper dermis small vessels. On immunohistochemistry, intravascular cells were CD 68+ and negative for estrogen and progesterone receptors, CK7, EMA and AE1/AE3 and endothelial cells were CD64+, leading to the diagnosis of intravascular histiocytosis.

## Introduction

Intravascular histiocytosis is a rare condition characterized by the presence of histiocytes within the dilated lymphatic vessels of the dermis. It can be primary or secondary to systemic diseases such as rheumatoid arthritis and metallic prosthesis. The pathogenesis of the disease is still unclear.

## Case report

**Figure 1:**
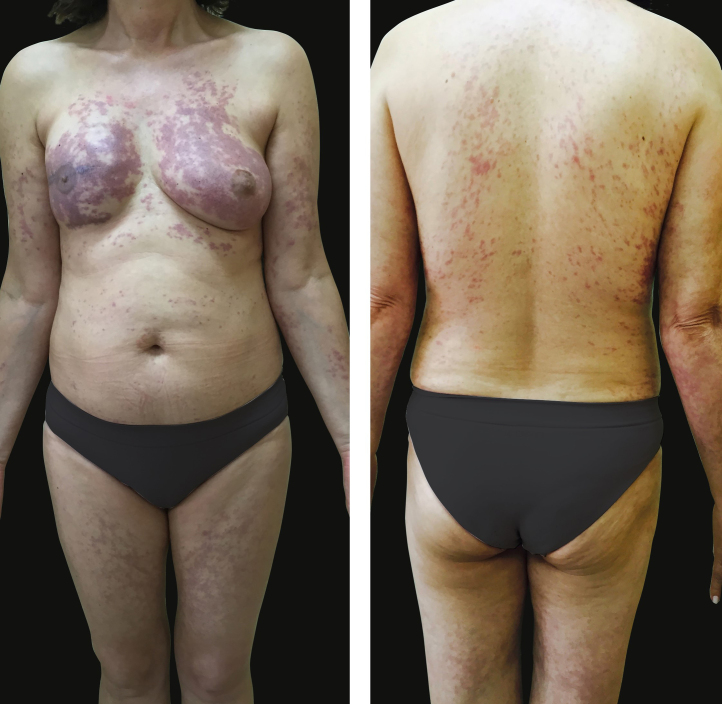
Erythematous/purple patches on the breasts, back and limbs.

**Figure 2:**
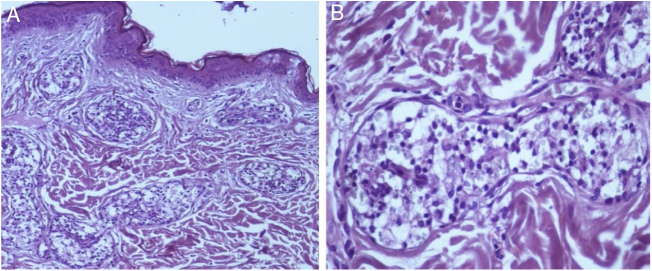
(A) Ectasia, thrombosis and recanalization of upper dermal vessels and the presence of histiocytoid cells inside of those vessels, (Hematoxylin & eosin, ×100). (B) Detail of the histiocytoid cells, (Hematoxylin & eosin, ×400).

**Figure 3:**
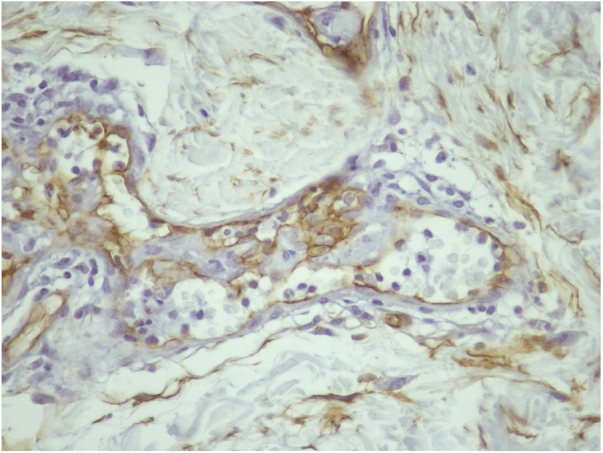
Immunohistochemistry CD 34 (400×): positive in the endothelial cells.

**Figure 4:**
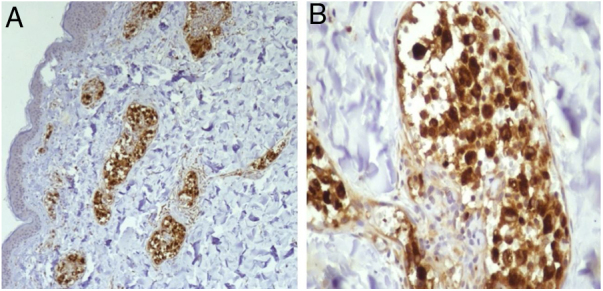
(A) Immunohistochemistry CD68, 200×: positive in the intravascular cells. (B) Detail of (A) (400×).

A 55 year-old female patient referred the onset of assymptomatic erythematous/purple patches in the breasts with progression to the back and proximal limbs one year and 6 months ago (Fig. 1). The patient presented with infiltrative ductal breast cancer in the right breast treated in 2006 and proceeded to the placement of silicone implant in 2009. The main clinical hypothesis were telangiectatic metastatic breast carcinoma and three cutaneous biopsies were performed in the left breast and one in the right thigh. The histopathology revealed ectasia, thrombosis and recanalization of upper dermal vessels and the presence of histiocytoid cells inside of those vessels (Fig. 2). The intravascular cells were negative for estrogen and progesterone receptors, CK7, EMA and AE1/AE3 in the immnunohistochemistry, which excluded the hypothesis of telangiectatic metastatic breast carcinoma. The endotelial cells were CD34 positive (Fig. 3). The CD68 antibody was then utilized and was positive in the intravascular cells, confirming the histiocytoid origin and leading to the diagnosis of intravascular histiocytosis (Fig. 4).

## Discussion

Intravascular histiocytosis is a rare condition first described in 1994. It is characterized by the presence of histiocytes within the dilated lymphatic vessels of the papillary and reticular dermis.^1^, ^2^ Since 2003, the disease has been included on the spectrum of reactive cutaneous angiomatosis, as well as reactive angioendotheliomatosis. Intravascular histiocytosis may be primary or secondary mainly to rheumathoid arthritis, but also to other inflamatory diseases such as Crohn's disease, dermatomyositis and monoclonal gammopathy, metallic prosthesis, chronic infections and neoplasms, specially breast cancer. However, there are cases showing association with colorectal cancer, melanoma, vulvar necrosis and osteoarthrosis.^1^, ^3^

The disease affects mostly women with 17–87 years old (65 years, mean age). The cutaneous lesions are most commonly limited and presented as erythematous/purple macules or pacthes or with livedo reticular like lesions in the limbs or trunck. They are rarely disseminated. In cases associated with rheumathoid arthritis, metallic prosthesis and osteoarthrosis, the lesion appeared near the joint and in cases associated with breast cancer, they appeared on the scar of the mastectomy.^1^, ^2^

The pathogenesis remains nuclear,^4^ however the findings suggest that the key to the pathogenesis can be the migration of histiocytes caused by the surrounding inflammatory reaction. Requena et al. postulated that intravascular histiocytosis would be caused by congenital or acquired lymphangiectasy or lymphatic obstruction, by trauma, surgery, radiation or infections.^2^, ^5^, ^6^ Other hypothesis is that chronic inflammation would lead to stasis and more exposition to antigens that would stimulate proliferation and aggregation of histiocytes within lymphatic vessels. The role of TNF is being taking in into account because of the disease's association with rheumathoid arthritis and other disorders associated with histiocytes, such as multicentric reticulohistiocytosis and intersticial granulomatous dermatites.^1^, ^3^ The histologic findings consist of CD68+ histiocytes aggregates within the dilated lymphatic vessels (podoplanin+) in the papillary and reticular dermis. Histophatological differential diagnosis with reactive angioendotheliomatosis remains controversial and some authors suggest that they represent different aspects of the same phenomenon in which the presence of histiocytes would lead to formation of microthrombos and endothelial proliferation.^2^, ^7^ Telangiectatic metastatic breast carcinoma is a rare form of cutaneous metastasis of breast cancer that consists in the onset of erythemathous macules with telangiectasias or lymphangioma circumscriptum. Histopathology reveals intravascular proliferation of tumoral cells that can be positive for estrogen and progesterone receptors.^8^, ^9^

The course of the disease is chronic and there is not a specific treatment. In the secondary cases it is necessary to treat the overlying disease. Some patients evolved into cure after removal of metallic prosthesis.^1^, ^2^, ^10^ Therapy with infliximab, methotrexate, pentoxifiline, radiotherapy, corticosteroids and topic tracolimus have variable response and frequent relapses.^1^, ^2^ Our case presented an exuberant disseminated disease and possible association with silicone breast implant, for its beginning in the breasts, which association has never been described in the literature.

## Financial support

None declared.

## Authors' contributions

Yasmin Gama Abuawad: Conception and planning of the study; elaboration and writing of the manuscript; obtaining, analysis, and interpretation of the data; critical review of the literature.

Ticiana de Andrade Castelo Branco Diniz: Conception and planning of the study; obtaining, analysis, and interpretation of the data; critical review of the literature.

Priscila Kakizaki: Approval of the final version of the manuscript; conception and planning of the study; obtaining, analysis, and interpretation of the data; effective participation in research orientation; intellectual participation in the propaedeutic and/or therapeutic conduct of the studied cases; critical review of the literature; critical review of the manuscript.

Neusa Yuriko Sakai Valente: Approval of the final version of the manuscript; conception and planning of the study; obtaining, analysis, and interpretation of the data; effective participation in research orientation; intellectual participation in the propaedeutic and/or therapeutic conduct of the studied cases; critical review of the literature; critical review of the manuscript.

## Conflicts of interest

None declared.

## References

[1] Barba E., Colato C., Girolomoni G. (2015). Intralymphatic histiocytosis: a case report and review of literature. J Cutan Pathol.

[2] Requena L., El-Shabrawi-Caelen L., Walsh S.N., Segura S., Ziemer M., Hurt M.A. (2009). Intralymphatic histiocytosis. A clinicopathologic study of 16 cases. Am J Dermatopathol.

[3] Bakr F., Webber N., Fassihi H., Swale V., Lewis F., Rytina E. (2014). Primary and secondary intralymphatic histiocytosis. J Am Acad Dermatol.

[4] Rhee D.Y., Lee D.W., Chang S.E., Lee M.W., Choi J.H., Moon K.C. (2008). Intravascular histiocytosis without rheumatoid arthritis. J Dermatol.

[5] Piccolo V., Ruocco L., Russo T., Baroni A. (2014). A possible relationship between metal implant-induced intralymphatic histiocytosis and the concept of the immunocompromised district. Int J Dermatol.

[6] Bidier M., Hamsch C., Kutzner C., Enk A., Hassel J.C. (2015). Two cases of intralymphatichistiocytosis following hip replacement. J Dtsch Dermatol Ges.

[7] Mazloom S.E., Stallings A., Kyei A. (2017). Differentiating intralymphatic histiocytosis, intravascular histiocytosis, and subtypes of reactive angioendotheliomatosis: review of clinical and histologic features of all cases reported to date. Am J Dermatopathol.

[8] Marneros A.G., Blanco F., Husain S., Silvers D.N., Grossman M.E. (2009). Classification of cutaneous intravascular breast cancer metastases based on immunolabeling for blood and lymph vessels. J Am Acad Dermatol.

[9] Shinohara M.M., Tozbikian G., Wolfe J.T., Shin S.J., Mies C., Elenitsas R. (2013). Cutaneous metastatic breast carcinoma with clear cell features. J Cutan Pathol.

[10] Tang M.M., Irla N., Simon D., Beltraminelli H., Borradori L. (2015). Cutaneous reactive angiomatosis with foreign body reaction after total knee replacement with a defective implant: a diagnostic challenge with a review of the literature. J Eur Acad Dermatol Venereol.

